# The report of ovarian tissue transplant in Iran: A case report

**DOI:** 10.18502/ijrm.v22i4.16393

**Published:** 2024-06-12

**Authors:** Fatemeh Anbari, Mohammad Ali Khalili, Mahboubeh Vatanparast, Saeid Haghdani, Maryam Eftekhar

**Affiliations:** ^1^Research and Clinical Center for Infertility, Yazd Reproductive Sciences Institute, Shahid Sadoughi University of Medical Sciences, Yazd, Iran.; ^2^Department of Reproductive Biology, Shahid Sadoughi University of Medical Sciences, Yazd, Iran.; ^3^Molecular Medicine Research Center, Rafsanjan University of Medical Sciences, Rafsanjan, Iran.; ^4^Andrology Research Center, Yazd Reproductive Sciences Institute, Shahid Sadoughi University of Medical Sciences, Yazd, Iran.

**Keywords:** Cryopreservation, Tissue transplantation, Leukemia, Vitrification, Ovarian follicle.

## Abstract

**Background:**

Cancer treatments such as chemotherapy and radiotherapy increase the chance of ovarian failure. Ovarian tissue transplantation (OTT) is a viable option for fertility preservation in these cases. We aim to report ovarian transplantation in a leukemia case undergoing the vitrification method.

**Case Presentation:**

The case was a 28-yr-old female in Research and Clinical Center for Infertility, Yazd, Iran who was suffering from leukemia. Ovarian biopsy was performed by laparoscopy surgery and transported to cryopreservation lab at 4 C for 1–2 hr. The ovarian cortex was removed from the medulla, and ovarian strips were cryopreserved by vitrification. This procedure used the equilibration and vitrification solutions including medium 199 supplemented with 20% serum, and ethylene glycol and dimethyl sulfoxide with concentrations of 7.5% and 20%, respectively. Before doing OTT, we assessed the tissue viability and follicular count by chick embryo chorioallantoic membranes and histologic survey, respectively. OTT was done after complete remission, following warmed tissue sutured together and transplanted on the residual medulla on the right side. On the left side, the ovary was removed completely; however, 2 strips were put on the peritoneal pocket. Anti-Müllerian hormone, follicle-stimulating hormone, and luteinizing hormone levels were 0.1 ng/mL, 36.5 mIU/mL, and 19.8 mIU/mL before OTT. During a 6-month follow-up, the anti-Müllerian hormone increased to 0.9, and then follicle-stimulating hormone and luteinizing hormone levels decreased dramatically until 17.47 mIU/mL and 6.71 mIU/mL, respectively. Also, the patient had 3 cycles of menstrual periods.

**Conclusion:**

We demonstrated an appropriate hormonal profile, and the restoration of the menstrual cycle might indicate a successful transplant. Further investigations are needed to achieve successful clinical outcomes.

## 1. Introduction

American Cancer Society recorded that nearly 1,000,000 women and 11,000 pre-puberty girls have been suffering from cancer (1). Cancer treatments such as chemotherapy, radiotherapy, and bone marrow transplantation are used for cancerous women and have led to an increased chance of survival rate. However, these factors are gonadotoxic agents and cause irreversible damage to the ovarian germ cells, depleting the ovarian reserve, and leading to premature ovarian failure (2).

Acute lymphoblastic leukemia (ALL) is involved with the degeneration of the lymphoid progenitor cells and a high risk of metastasis (3). By understanding the challenges posed by leukemia and chemotherapy-induced infertility, we can appreciate the importance of fertility preservation techniques in providing hope and options for future reproductive success. Ovarian tissue cryopreservation (OTC) offers a viable option for fertility preservation in women diagnosed with leukemia or undergoing chemotherapy (4). OTC and subsequent ovarian tissue transplantation (OTT) also offer benefits including protection against early menopause and long-term fertility preservation. It could be applied to women at a suitable age with adequate ovarian reserve, as well as patients who cannot delay their cancer treatment due to controlled ovarian stimulation (5). Vitrification is one of the proposed methods that has been recently noticed as an optimal technique for preserving ovarian tissue (2, 6). According to the latest report, frozen and thawed ovarian slices resulted in about 200 live births (7).

In Iran, the birth live after OTT has not been reported yet. Here, we aim to report an orthotopic ovarian transplantation in ALL cases undergoing the vitrification method in Research and Clinical Center for Infertility, Yazd, Iran.

## 2. Case Presentation 

### The case study

The case was a 28-yr-old woman in Research and Clinical Center for Infertility, Yazd, Iran, suffering from ALL in 2016. She started chemotherapy with 30 mg vincristine and 975 mg Adriamycin for 6 months and then referred for OTC before the hematopoietic stem cell transplantation in May 2017. Before the OTC procedure, anti-Müllerian hormone (AMH) was also checked, which was within the normal range (3.66 ng/mL).

### Ovarian tissue preparation

The ovarian biopsy (nearly 3 × 1.5 cm^2^) was removed during laparoscopic surgery and transported to our laboratory on crushed ice for 1–2 hr. The transferring medium was phosphate-buffered saline (PBS, Medicago AB, Uppsala, Sweden) supplemented with 5% human serum albumin (HSA, GIBCO/BRL, Germany).

### OTC

**Figure 1:**
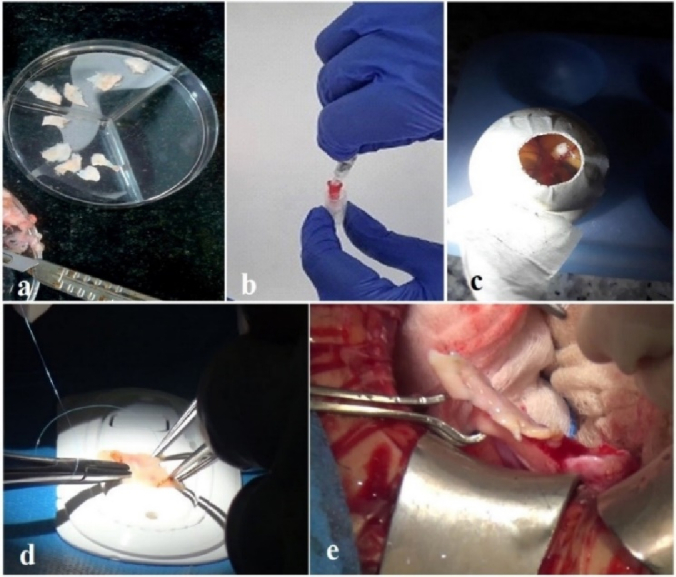
The OTC process: A) Ovarian tissue slicing by the surgical blade, B) The needle immersion vitrification methods for OTC. Both needles and the inserted tissue were placed into the cryovials, C) Warmed ovarian tissue viability confirmation, by the chick embryo chorioallantoic membranes transplantation, D) Ovarian tissue strips sutured to create a unit texture, E) Ovarian tissue autotransplantation.

After the oocyte evaluation, the ovarian tissue was prepared for OTC (Figure 1). In this aim, the ovarian cortex was removed from the medulla, and 11 ovarian strips in size 1 × 1 × 5 mm were prepared, using a 22-gauge surgery scalpel (Figure 1a).

Our institute policy for OTC was vitrification, with some modifications (8). The handling medium (Medium 199, Sigma-Aldrich, Germany) was supplemented with a 20% synthetic serum substitute (SSS; Irvine Scientific, USA). 2 equilibration (ES) and vitrification solution (VS) contained ethylene glycol and dimethyl sulfoxide (DMSO, Sigma-Aldrich, Germany) (7.5%, 7.5% in ES, 20%, and 20% in VS, respectively). VS also contained 0.5 mol/l sucrose. ES was done on ice water (4 C, 25 min), followed by VS (15 min). In the final step, the needle immersion method was used to immerse the tissue in liquid Nitrogen (Figure 1b).

The warming steps were performed through the different concentrations of sucrose, in the handling medium, the first was 1.0 mol/l sucrose (1 min), the second contained 0.5 mol/l sucrose (5 min), and the third was twice washing of the strips in the handling medium (10 min, RT). Before transplantation, the warmed tissues were incubated for 45 min in α-minimum essential medium, in a humidified incubator (37 C).

### Tissue viability confirmation

As the OTC was not a routine technique in our institute, and it had not been done before 2015, chick embryo chorioallantoic membranes (CAM) were used (in the first 20 cases) to ensure tissue viability after cryopreservation. However, CAM hurts the follicle ultrastructure and is a valid technique for confirmation of ovarian tissue viability after warming (9).

With this aim, 1-day-old eggs were transferred to the laboratory at a temperature of 15 C and incubated at 37 C and a humidity of 65%. After that the eggshell was removed from the fertilized egg on day 3 of incubation, and one square of the warmed tissue ( ∼ 1 × 1 × 1) was placed on the CAM surface (the site of large vessels) (Figure 1c). If the removed tissue was pink and well-vascularized, after 5 days, it was considered alive after tissue cryopreservation (9).

**Figure 2:**
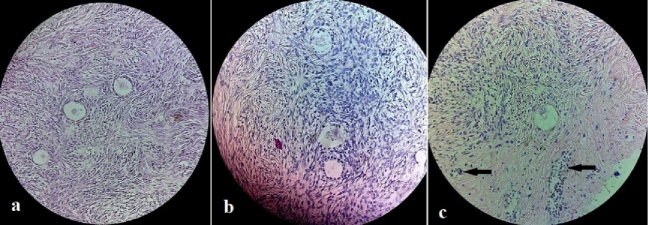
H&E staining of the transplanted tissue: A) Good morphology follicles in the fresh ovarian tissue, B) Follicles after the ovarian tissue vitrification, and C) One healthy primordial follicle, in the border between ovarian stroma and CAM, after vitrified/warming and CAM transplantation. Avian vessels are distinguished by nucleated erythrocytes (black arrows).

In all cases of the fertility preservation program, one of the fresh, thawed strips went for histologic evaluation. Moreover, this will be a basic reference for future analysis, in this histologic survey, the presence of follicles was checked (an estimation of the ovarian reserve). The follicular count was 7.48 and 3.76 in the fresh and the thawed ovary, respectively. Also, after CAM removal, H&E staining was done for the transplanted tissue (Figures 2a, b, c).

As shown in figure 2c, one healthy primordial follicle can be found at the border between the ovarian tissue and CAM, where the CAM loose tissue invades the stroma. In this figure, the avian vessels can be distinguished from the nucleated erythrocytes (black arrows). Immunohistochemistry evaluation and CD45 antibody were also observed by a pathologist, and no evidence of leukemia cell infiltration (for 3 stages of fresh, vitrified, and CAM transplanted tissue) was observed.

### OTT

After 6 yr, in August 2023, when she was in complete remission, she requested to transplant her tissue. Follicle-stimulating hormone and luteinizing hormone levels were 36.5 mIU/mL and 19.8 mIU/mL, respectively, and AMH levels were 0.1 ng/mL without any follicles in the pelvic ultrasound assessment.

At the time of operation, the vitrified tissue was delivered to the operating room for transplantation. The individual tissue strips are quilted together with 8–0 nylon (Figure 1d). At first, the removal of the dead cortex was done from the right ovary under laparotomy. The thawed OT sutured on the residual medulla. On the left side, the ovary was removed completely; however, 2 strips were put on the peritoneal pocket (Figure 1e). During a 6-month follow-up, the patient had 3 cycles of menstrual periods. At first, AMH increased to 0.9, and then follicle-stimulating hormone and luteinizing hormone levels decreased dramatically to 17.47 mIU/mL and 6.71 mIU/mL, respectively. Follicle growth was observed using ultrasonography.

### Ethical considerations

She was informed about the complications of surgery and the success rate of this procedure, and the consent form was signed by her.

## 3. Discussion

This is a case report that shows appropriate ovarian cryopreservation and transplantation methods in terms of improving hormonal conditions and starting the menstrual cycle at the Research and Clinical Center for Infertility, Yazd, Iran.

In this case, the slices of tissue were vitrified. Although the vitrification procedure is an establishment method in oocyte and embryo cryopreservation, in OTC the procedure is controversial. However, it has been proven that slow freezing method employs controlled cooling rates to prevent ice crystal formation, which can damage cells and tissues. On the other hand, vitrification minimizes ice crystal formation by rapidly cooling the tissue to ultralow temperatures, forming a vitrified state that avoids the creation of damaging ice crystals in OT. This technique stands out due to reduced handling time, and inexpensive equipment. Also, it has potential benefits in preserving cellular integrity and viability during the freezing and thawing processes (10).

Some studies evaluated the effect of vitrification on morphology, viability, and DNA damage in OT (11, 12) no significant differences were noticed in morphology and distribution of follicular between the 2 methods. According to a review study, 6 studies have addressed the issue of comparison of DNA fragmentation or nuclear chromatin, of which 3 studies reported no difference between the 2 groups. 2 studies reported higher levels of DNA fragmentation in tissue undergoing slow freezing, and one was found in vitrified/warmed tissues (2). Kagawa and co-workers reported a high oocyte viability rate (89%) in vitrified/warm tissue similar to fresh human samples (8). One of the vitrification procedures is called needle immersion vitrification (NIV). It showed that NIV can preserve the structure of primordial follicles and stromal cells in both human and mouse models (13). In our study CAM transplantation and histological evaluation presented high viability after the NIV method. In the context of OTT, around 70% of follicles are impacted by ischemia until the process of revascularization. Therefore, the optimization of ovarian trimming for obtaining the right thickness of OT, ideal OTC processes, effective approaches for enhancing follicle viability, and the best site of transplantation can improve the revascularization of OT (14, 15).

The success of the ovarian tissue transplant operation strongly depends on the age and preservation of the ovarian tissue (16). It should be noted that ALL cases must receive at least a single dose of chemotherapy before OTC. This is due to the high risk of malignant cells in untreated leukemia (17). Poirot and co-workers showed that the duration of ovarian activity was decreased in women who received prior chemotherapy during OTC, but the pregnancy rate was not less (18). In our case, the patient had received several doses of chemotherapy for a long time, and even though the ovarian reserve was in the normal range, it could have a negative effect on transplant outcomes. In general, fertility preservation has paramount importance for ALL patients, because it happens at a young girl and needs immediate gonadotoxicity of the conditioning regime with the possibility of an 80% risk for premature ovarian failure. Applying assessment strategies such as histology and immunohistochemistry, FISH, next-generation sequencing, and xeno-transplantation is suggested to detect ovarian tissue contamination by leukemic cells (4, 19). In this case, the report of immunohistochemistry and CD45 markers were negative before OTT.

## 4. Conclusion

In conclusion, we demonstrated a promising OTT and successful vitrification program in ALL patients. After tissue warming, the morphology and viability of follicles were confirmed by histologic evaluation and CAM transplantation. An appropriate hormonal profile and the restoration of the menstrual cycle might indicate a successful transplant. Further investigations are needed to achieve successful clinical outcomes.

##  Data availability

Data supporting the findings of this study are available upon reasonable request from the corresponding author.

##  Author contributions

Fatemeh Anbari, Saeid Haghdani, and Maryam Eftekhar designed the study and conducted the research. Mohammad Ali Khalili and Mahboubeh Vatanparast monitored, evaluated, and analyzed the results of the study. Fatemeh Anbari and Maryam Eftekhar had full access to all of the data in the study and takes responsibility for the integrity of the data and the accuracy of the data analysis. Further, authors reviewed the article. All authors approved the final manuscript and take responsibility for the integrity of the data.

##  Conflict of Interest

The authors declare that there is no conflict of interest.
