# Corrigendum to “The report of ovarian tissue transplant in Iran: A case report” [Int J Reprod BioMed 2024; 22: 323–328]

**DOI:** 10.18502/ijrm.v22i9.17481

**Published:** 2024-11-14

**Authors:** Fatemeh Anbari, Mohammad Ali Khalili, Mahboubeh Vatanparast, Saeid Haghdani, Maryam Eftekhar

The publisher has been informed of an error that occurred on page 323 in which the third authors affiliation must be changed to Molecular Medicine Research Center, Research Institute of Basic Medical Sciences, Rafsanjan University of Medical Sciences, Rafsanjan, Iran. On behalf of the author, the publisher wishes to apologize for this error. The online version of the article has been updated on September 30, 2024.

